# Supplementary figures and images for: Drug repositioning for pan-cancers of the digestive system: Identification of amonafide and BX795 as potential therapeutics via integrative Omics analysis
Source: PLoS One. 2025 Jun 16;20(6):e0325700. doi: 10.1371/journal.pone.0325700 (PMC12169532; doi:10.1371/journal.pone.0325700)

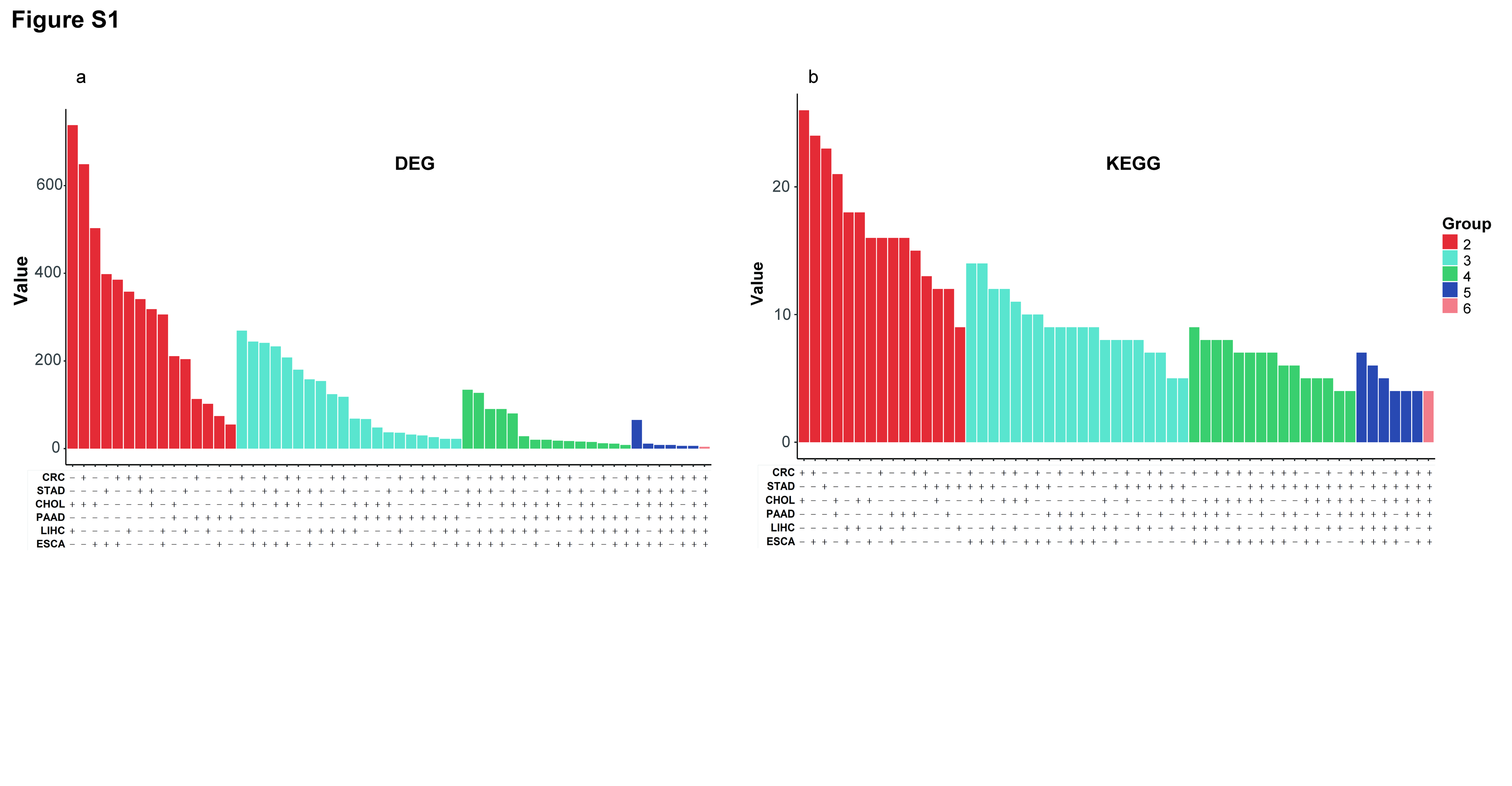

Supplement: S1 Fig — (TIF) [file pone.0325700.s001.tif]

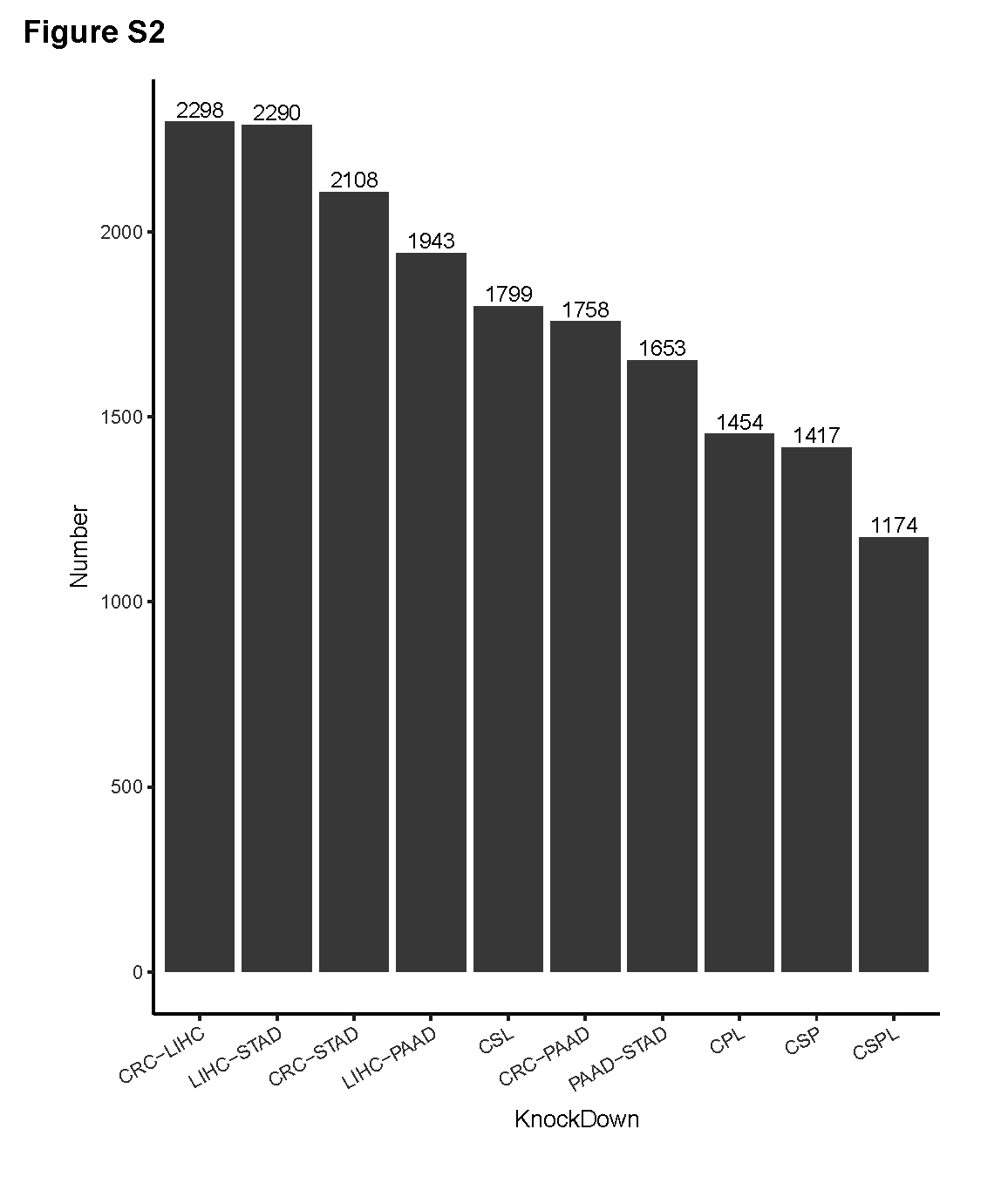

Supplement: S2 Fig — (TIF) [file pone.0325700.s002.tif]

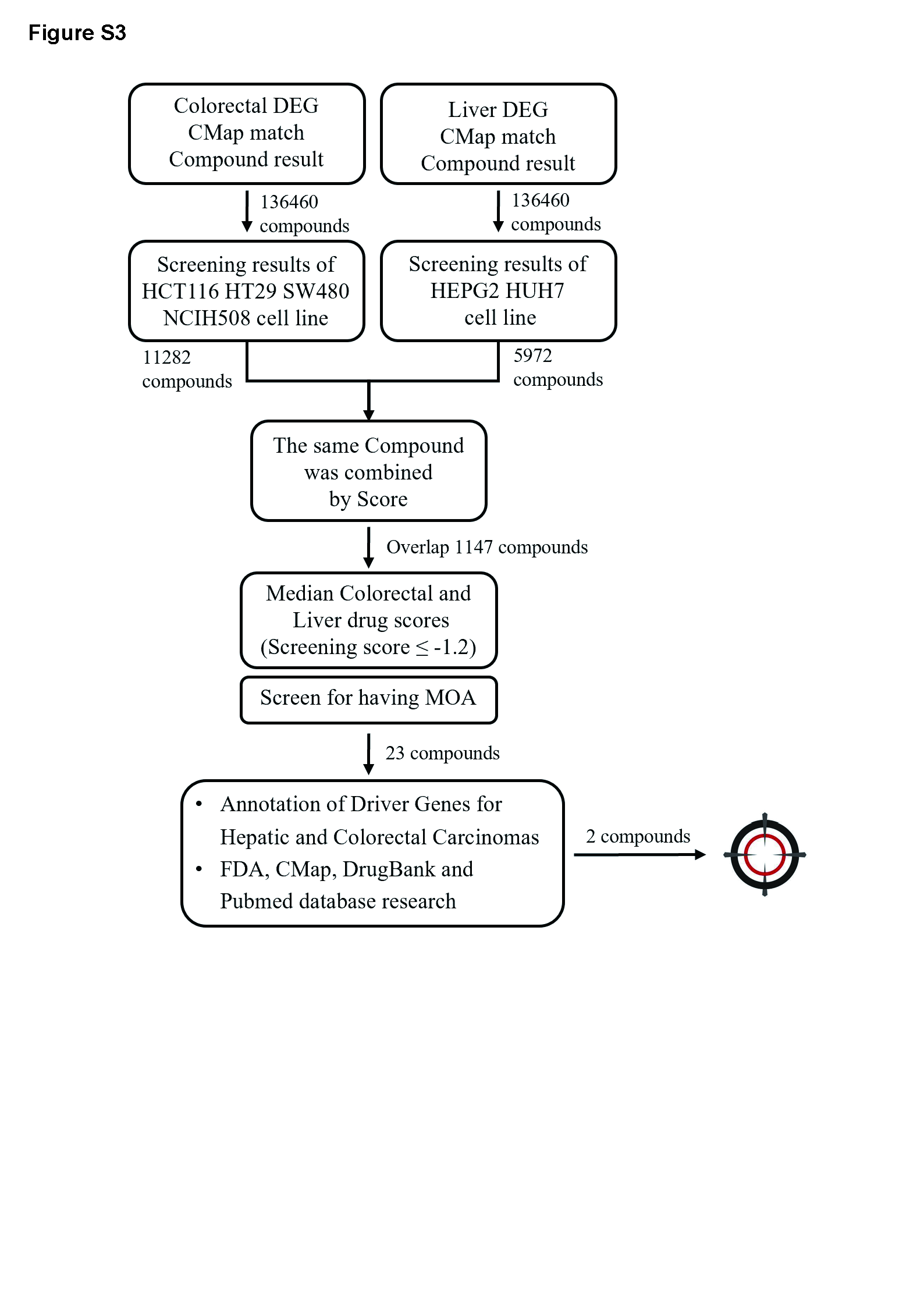

Supplement: S3 Fig — (TIF) [file pone.0325700.s003.tif]

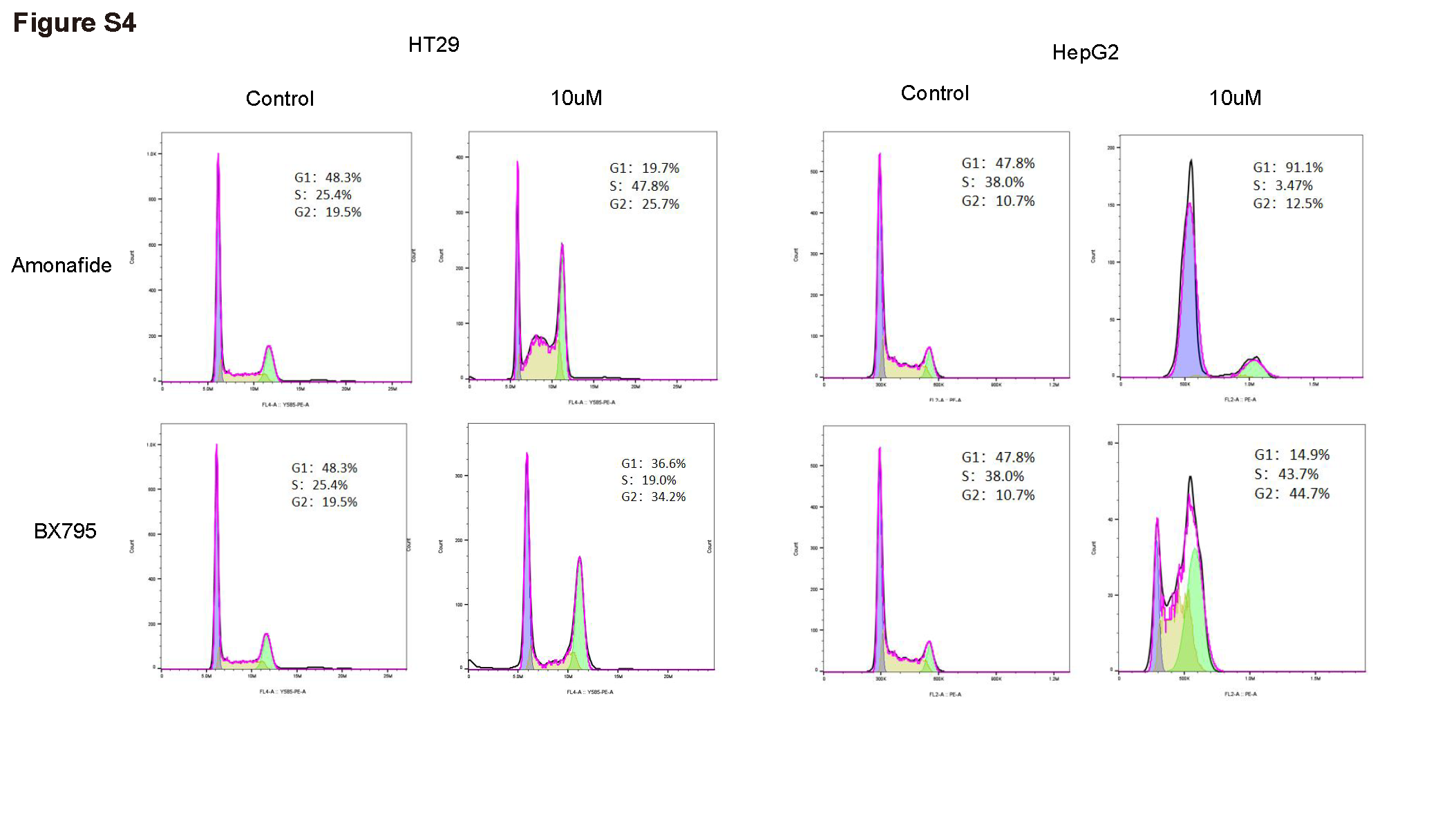

Supplement: S4 Fig — (TIF) [file pone.0325700.s004.tif]

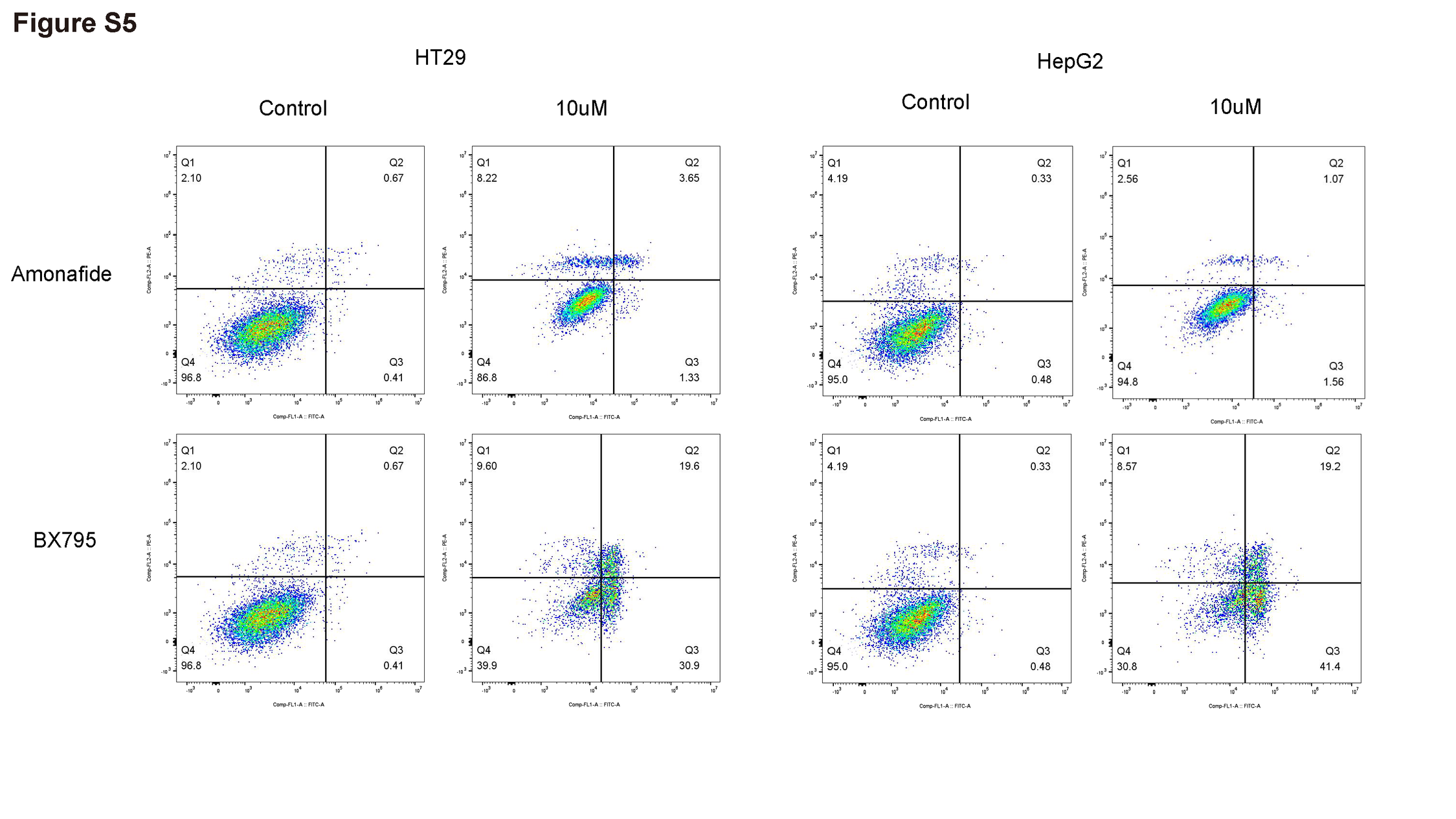

Supplement: S5 Fig — (TIF) [file pone.0325700.s005.tif]

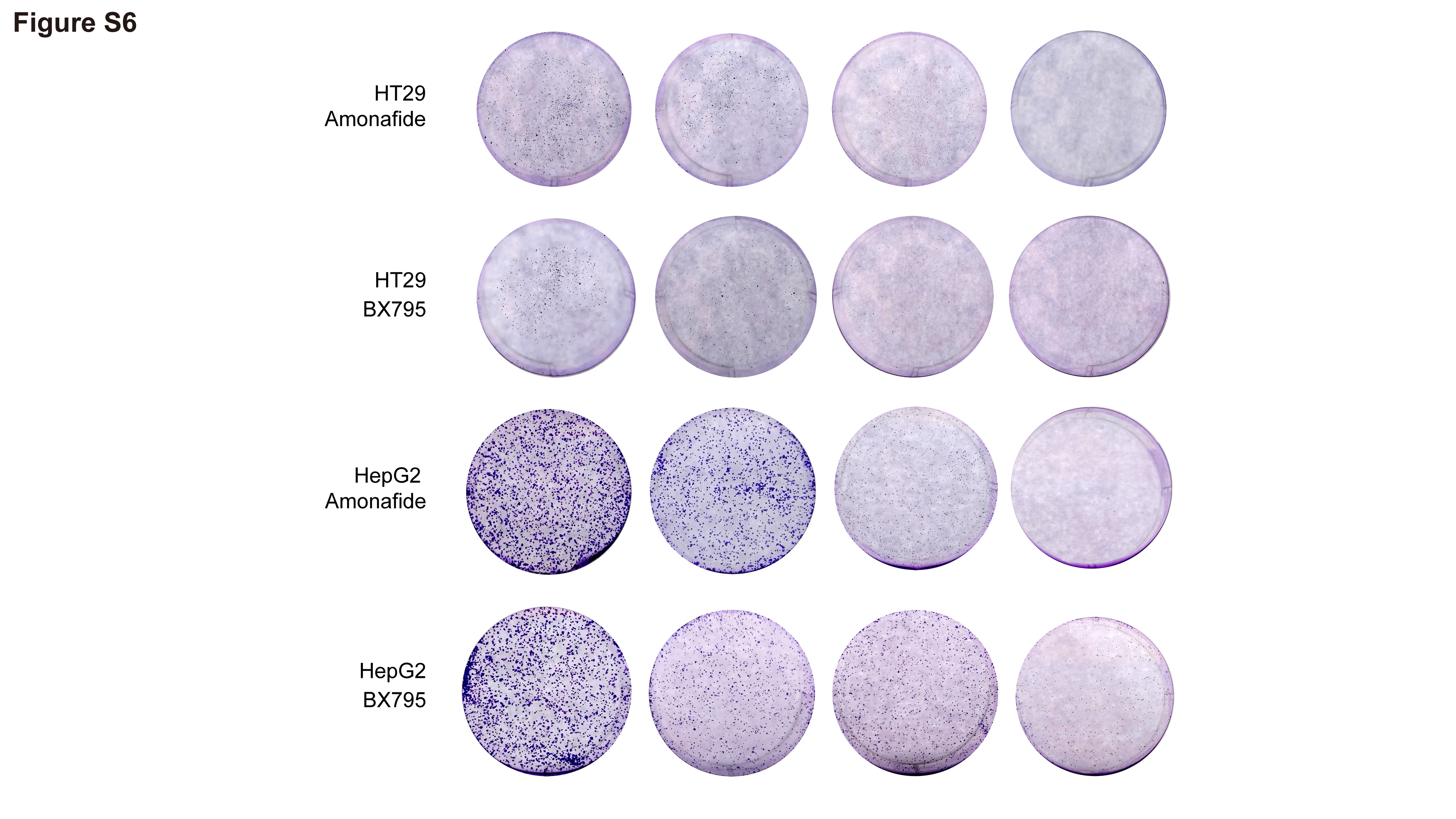

Supplement: S6 Fig — (TIF) [file pone.0325700.s006.tif]
