# Supplementary material for: Drug repositioning for pan-cancers of the digestive system: Identification of amonafide and BX795 as potential therapeutics via integrative Omics analysis
Source: PLoS One. 2025 Jun 16;20(6):e0325700. doi: 10.1371/journal.pone.0325700 (PMC12169532; doi:10.1371/journal.pone.0325700)
Supplement: Attachment — Submitted filename: Letter -Response to Reviewers.docx [file pone.0325700.s016.docx]

**Letter to the Editor**

**Subject: Revise of Manuscript for Publication Consideration**

Dear Editor,

**Enclosure Notification**

Thank you for the opportunity to revise our manuscript titled “Drug Repositioning for Pan-Cancers of the Digestive System: Identification of Amonafide and BX795 as Potential Therapeutics via Integrative Omics Analysis” in response to the comments and suggestions provided by the reviewer. We appreciate their valuable feedback and have made significant improvements to address their concerns.

We have carefully considered each comment raised and have made the following revisions to the manuscript:

Response to Reviewer 1:

- Question 1: Important data for study analyses were obataind form the TCGA databse. However, considering the patway of LIHC, it is well known that the etiology of underlying liver disease (viral, non-viral, meabolic, autoimmune chronic liver disease) may affect the cancer development and patway. Therefore, if available, it would be of major relevance to report the etiology of enrolled LIHC patients.

- Revision: This is a very good suggestion. This is the deficiency of this article, which lacks a more detailed analysis of liver disease types. Moreover, the clinical information for specific patients in TCGA is incomplete and therefore cannot be provided.

- Question 2: Of interest, the authors found that the core proteins of NXF1 and NCBP2 in the Ubiquitin Mediated Proteolysis pathway, and RPS27A as a core protein in RNA Translation pathway, were also drive genes of CRC and LIHC and these pathways may hold the potential for targeted therapeutic intervention that leverages the shared molecular mechanisms of the two cancers. Importantly, capecitabine, a prodrug of 5-FU has anti-tumor efficacy not only in CRC but also has demonstrated a good anti-tumor efficacy in hepatocellular carcinoma patients failing sorafenib therapy as recently demonstrated (doi: 10.1007/s00432-017-2556-6.). This may represent a further proof of common therapeutic target(s) and is therefore worth to be recalled and discussed.

- Revision: It has been added in lines 53-55.

Response to Reviewer 2:

- Question 1: Were toxicity assays performed on normal epithelial cells, such as FHC for colon or THLE-2 for liver?

- Revision: This article did not conduct toxicity assays. The candidate drugs were approved for marketing by the FDA, and experiments on the corresponding cells were also mentioned in the references. We will pay more attention to this aspect in the subsequent research.

- Question 2: Could long-term treatment induce resistance (e.g., via ABC transporters)?

- Revision: This is a very good suggestion. However, this article aims to discover new drugs through drug reuse. In subsequent studies, we will pay more attention to this aspect.

- Question 3: The authors must State whether replicates were technical or biological (e.g., n = 3 independent experiments

- Revision: Biological repetition, mentioned in the Method section 185; Line 194, etc.

- Question 4: Compare drug efficacy to standard therapies (e.g., 5-FU for CRC, sorafenib for LIHC).

- Revision: This is a very good idea. Due to the large number of cell experiments in this article and time constraints, it is impossible to supplement. This limitation has been pointed out in the subsequent discussion section.

Line 440，Moreover, our research lacks Compare drug efficacy to standard therapies, but these drugs do have inhibitory effects on two types of cancers.

- Question 5: Figure 1: UMAP/hierarchical clustering suggests homogeneity, but the text later highlights CRC-LIHC differences. Clarify this discrepancy!

- Revision: Because UMAP/ hierarchical clustering was analyzed based on the whole transcriptome, it showed that different cancer tissues were consistent as a whole. However, in the subsequent differential genes and gene expression modules, it was demonstrated that there were differences in CRC-LIHC, and common regulatory modules were found.

Response to Reviewer 3:

- Question 1: Introduction: I suggest a more in-depth discussion of the current need to find therapies for cancers in general, and then focus on gastric cancer. It would be beneficial to include more detail on the impact of current treatments and how drug repositioning could address these issues. In addition, they might consider mentioning the economic benefits of drug repositioning in oncology.

- Revision: It has been added in lines 45-61.

- Question 2: Results: I suggest going a little deeper into the results obtained using the Connectivity Map (C-Map) and the impact of the small molecules identified. This would help to provide a greater understanding of how these results could be applied in clinical settings.

- Revision: All the matched obtained small molecules and their target genes are mentioned in Supplementary table S8, and some literature on the role of small molecules in cancer research is provided.

- Question 3: Discussion: I propose to further address the clinical impact of the findings of this research. It would be useful to explore how the compounds Amonafide and BX795 could be integrated into combination or alternative treatments. In addition, it would be valuable to discuss the limitations of the in vitro experiments performed and possible directions for future in vivo validations. I also suggest including an analysis of the economic benefits of drug repositioning and its potential impact on the pharmaceutical industry and correlate this with their findings.

- Revision: It has been added in lines 375-391.

- Question 4:  Conclusions: I recommend subtly adding a mention of the prospects towards clinical trials, to highlight the relevance of the results obtained and their possible future application in clinical practice.

- Revision: It has been modified on line 453-455. These findings provide a foundation for future research and these compounds are known clinical research drugs. Drug repurposing may change the treatment prospects for patients with these diseases.

Thank you for your time and consideration. We look forward to your positive response.

Sincerely,

Fei Gao, Ph.D.
Principal Investigator
HIM-BGI Omics Center
Zhejiang Cancer Hospital
Hangzhou Institute of Medicine (HIM)
Chinese Academy of Sciences (CAS)
Hangzhou, China
Email: [flys828@gmail.com]
